# Towards a radical highway geography: Berlin and the remaking of city logistics in global capitalism

**DOI:** 10.1177/0308518X251361632

**Published:** 2025-08-26

**Authors:** Susanne Soederberg

**Affiliations:** Global Development Studies, Queen’s University, Kingston, ON, Canada

**Keywords:** Radical geography, city logistics, Berlin, European Union, German state, Highway A100, state scales, road freight sector

## Abstract

Highways are vital to global supply chains, enabling the dominant form of circulating goods inland by truck. Within critical economic geography and related disciplines, however, insufficient attention has been placed on developing a radical highway geography that positions highways within the evolving relationships between global capital, state scales and the labour of moving goods. I fill this silence by applying a historical-geographical materialist lens to Germany’s most congested, costly, and controversial highway – Berlin’s intercity A100 – to explore the entanglements of highways, labour power and the capitalist state within the socio-spatial and temporal dynamics of global capitalism. By following the A100 from the 1950s to the proposed completion of its contentious 16th extension in 2025, I argue that the 16th construction phase is the outcome of continual attempts by the capitalist state – at various scales of intervention – to annihilate space through time. These time–space compressions, which are incomplete, contradictory and contested, facilitate the circulation of commodities – understood here as urban freight and labour power – across space more rapidly and at lower cost, leading not only to a remaking of city logistics but also in the embodied labour of truck drivers, whose working lives increasingly reflect the pressures of accelerated circulation.

## Introduction

Highways are vital to global supply chains. With a majority of goods moved inland along highways by trucks, they are intrinsic to patterns of production and consumption in capitalism. As a consequence, highways invariably have a darker side. Aside from ecological violence linked to CO2 emissions, truck drivers belong to some of the most exploited workers in the global supply chain. Despite their importance in shaping daily life, highways have been widely neglected in critical economic geography and related disciplines, with insufficient attention placed on the labour that traverses them and the state scales that construct and regulate them. This oversight is mirrored in the handful of highway studies about resistance (Savitzky and Cidell, 2022), financialization (McManus and Haughton, 2021), mobility (Sheller, 2018), repurposing and removals (Stehlin, 2023). To fill this silence, I draw on the commitment of radical geography to *defetishize* our world (Christophers, 2011; Harvey 1990; Ouma, 2023) as a means to expose ‘powerful, important, and disturbing’ connections between highways, truck drivers and capitalist state scales that often remain hidden from view (Cook, 2004: 642).

**Figure 1.**
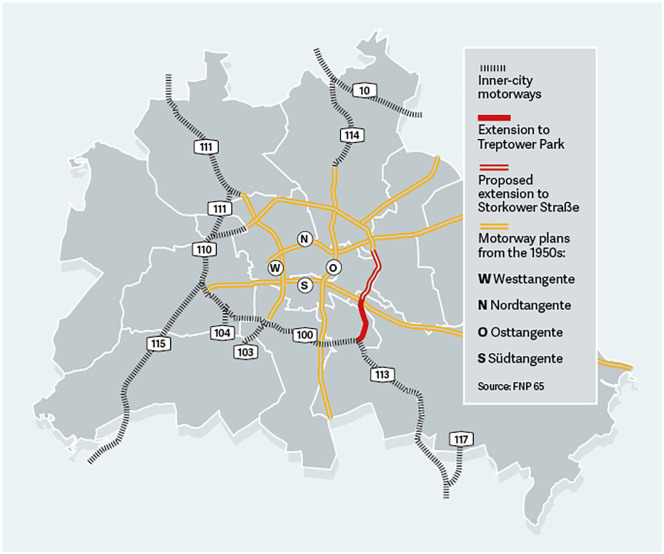
16th Extension of A100 – Dreieck Neukölln to Treptower Park. Source: The Berliner (2023).

I expose these connections by applying a historical-geographical materialist (HGM) lens to the case of Germany’s most contentious highway: the Autobahn 100 (hereafter: the A100). Located in Berlin, the 21-km intercity highway is the busiest highway in the country. Most of the vehicles that traverse the A100 are commuters and trucks. The latter are the backbone of Europe’s supply chains. Commercial road transport, for instance, hauls over 77% of inland freight across the continent (IRU, 2024). And, as the logistics hub of Europe, Germany and its highways play a vital role in moving goods along the continent’s global supply chains. Despite widespread protests, the federal state proceeded with a 16th extension of the A100 in 2013 to reduce travel time. This highly contentious 16th phase extends the highway 3.2 km eastward, from the district of Neukölln to Treptower Park (see Figure 1). Since 2013, construction costs have surged to over €720 million – equating to approximately €246,000 per metre – earning the A100 the dubious distinction of being the most expensive highway project in Germany history (RBB24, 2024).

**Figure 2.**
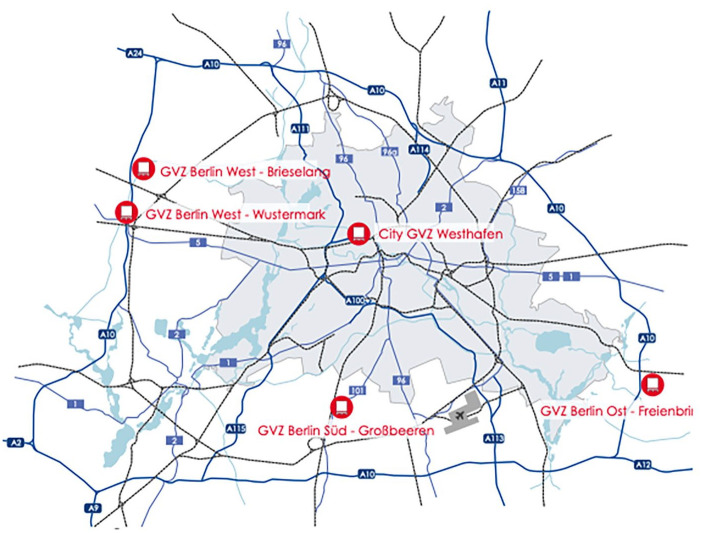
Berlin’s International Freight Centres (GVZs). Source: Berlin Business Location Centre. ‘Freight Distribution Centre GVZ’ (https://www.businesslocationcenter.de/en/infrastructure/freight-distribution-center-gvz).

Examining what lies beneath the surface of the A100 – beyond asphalt and concrete – reveals how the production of space and time underpins the everyday functioning of capitalism. It allows us to ask how highways, truck drivers, and state scales might be critically connected to offer greater insight into class-based contestation, contradictions and conflicts. Urban freight does not move itself into and across the congested intercity A100. These commodities require another commodity – the labour power of truck drivers – to transport them as quickly and cheaply as possible from manufacturing plants and intermodal terminals (ports) to international freight centres (*Gütervehrkehrzentrum* or GVZ; see Figure 2). GVZs describe ‘logistics centres, where the cargoes from different transport modes can be reloaded, compiled and prepared for transportation’ (DGG, 2024; Hesse, 2008; Klauenberg et al., 2020). Logistics companies and subcontracting firms presently act as the capitalists who hire road hauliers to facilitate this rapid movement. While the intercity A100 and truck drivers play an important role in moving an ever-greater demand for goods across a growing expanse of inland space at ever-faster speeds, they do not achieve this independently. Capitalist states are crucial to financing, planning, constructing and maintaining highways as well as regulating the road freight sector, including, my focus in this paper, its labour power. These state mediations – which are far from smooth – occur across a variety of scales. The European Union (EU), for instance, exercises authority over road freight, including drivers, federal highways such as the A100 falls under the jurisdiction of the German state, whereas the Berlin Senate is responsible for ensuring faster flow of goods and people through its ability to re-make city logistics. This exemplifies what Cowen (2014: 181) describes as the restructuring of already existing urban space into ‘more controlled and efficient spaces of circulation’. Two clarifying points are essential here. First, the reconfiguration of these urban spaces of circulation takes place beyond the city itself, within the broader dynamics of global capital accumulation (Angelo and Wachsmuth, 2015). Second, highway infrastructure and city logistics are entangled in mutually reinforcing, yet paradoxical, ways. As Harvey (1999) reminds us, the spatial mobility of commodities depends on the construction of transport networks that are fixed in space.

Combining my HGM lens and fieldwork, comprised of archival work and semi-structured interviews (~25) with various state and non-state organizations undertaken in Berlin and Brussels in 2024 and 2025, I argue that the 16th construction phase is the outcome of ongoing attempts by the capitalist state – at various scales of intervention – to annihilate space through time thereby reshaping the social relations of capitalism (Harvey, 2001). These time–space compressions, which are incomplete, contradictory, and conflict-ridden, facilitate the accelerated circulation of commodities – understood here as both urban freight and labour power – across space more rapidly and at lower cost. This acceleration is materially inscribed not only in the successive extensions of the A100 but also in the intensified labour of truck drivers, whose working conditions are stretched to accommodate the faster and farther flows of goods through the urban region.

My essay is organized in three main sections. In Section One’, I situate my study in relevant debates and outline the three analytics that comprise my HGM lens: highways as fixed capital, labour power, and capitalist state scales. In Section Two, I follow the A100 in 5 parts reflecting the timeline of the 16 extensions of the A100 from its opening in 1958 to its proposed completion in autumn of 2025. In Section Three, I summarize my argument, highlight several key insights, and discuss the political implications of a radical highway geography.

## Towards radical highway geography

### Context and silences

My HGM analysis of the A100 straddles two debates about highways and the circulation of goods: economic geographies of transport and critical logistics studies. In the early 2000s, a small group of economic geographers attempted to move beyond the dominant treatment of transportation infrastructure as technical and physical artefacts. With their focus on space (Hesse, 2008; Hesse and Rodrigue, 2004), these scholars set out to study highways – and other forms of transport infrastructure – as central features of urban logistics (Hall et al., 2006; Rodrigue, 2006). Working with a different set of questions than radical geography, these contributions are not concerned with the junctures between urban logistics in the global dynamics of capital accumulation and thereby investigating the root causes of the crises and conflicts involved in circulating an ever-growing demand for stuff at an ever-quicker pace. The radical perspective of circulation was addressed by a second debate aimed at grasping ‘how logistical systems function to coordinate, capture, and control the vicissitudes of daily life’ in global capitalism (Chua, 2022: 1443; Cowen, 2014). While these so-called critical logistic scholars have acknowledged the significance of infrastructure in the circulation of commodities (Chua et al., 2018; Coe, 2020; Danyluk, 2018) in capital accumulation, there remain at least two analytical areas that have been muted in these discussions. First, sparse attention has been paid to the historical connection between highways and truck drivers, who traverse these routes in the (re-)making of city logistics (Bonacich and Wilson, 2008, Viscelli, 2016). Second, there has been an under examination of the role and nature of the capitalist state, especially its multi-scalar spatial and temporal restructurings. The latter have, as I argue here, continued to shape profoundly the urban spaces of city logistics (cf. Chua, 2022).

### Radical reframing: Connecting the A100, labour power and capitalist state scales

#### Highways

My HGM reframing begins with a reconceptualization of the urban highway A100 as a special type of fixed capital in the global processes of capital accumulation (Angelo and Wachsmuth, 2015; Harvey, 2001). Unlike machinery and other means of production, highways represent a special type of fixed capital that operate as a physical framework for realizing value (Harvey, 1999). Examining highways in this way allows me to study the crises, contradictions and class dynamics at play in realizing surplus value across time and space through circulation (Harvey, 1999). The faster commodities reach their final site of consumption (purchase), the higher the profit rate for the producer, as less money is paid on transportation expense and the quicker the turnover time (Cowen, 2014; Harvey, 1990). On this view, congestion along strategic highways, such as the A100 poses a major issue for capital. The A100, for instance, plays a crucial role in Berlin’s urban freight network, as it serves as a key artery to long-distance, regional and urban road networks in the German capital connecting various districts and providing access to major logistics hubs (Klauenberg et al., 2020). The A100 has served as a major barrier to capital accumulation as it continues to slow the speed at which commodities – goods and labour power – flow across and into Berlin. Dealing with the congestion is complicated because as fixed capital the A100 cannot be moved without being destroyed (Harvey, 1990). As I discuss below, the response by the federal state has been to attempt to annihilate space through time by continually extending the A100. Viewing highways as historical social relations offers an important analytic for understanding the drive toward faster circulation of commodities across space. Equally important is theorizing the labour power that navigates these motorways – this constitutes my second analytic.

#### Labour power

For freight to traverse highways across ever-expanding spaces of capital, embodied in the widening of the European internal market, quickly and efficiently requires the labour power. Drivers of freight transport are a necessary component of the physical framework for value production embodied in highways. Decreasing transportation costs depends not only on the shorter circulation times of road freight facilitated by congestion-free highways. It also involves the wages remunerated to workers driving this freight and their labour time. As my study reveals, after many decades of deregulating the road freight sector, drivers’ wages have generally decreased whilst their working day has increased (Wahl and Weirich, 2023). Many of truck drivers operating in Germany are based in Central and Eastern European (CEE) Member States, with Poland leading the sector. Under the current arrangements, for example, drivers posted from CEE Member States to work in Western Member States such as Germany are paid far lower wages and are subjected to poor working conditions, often spending longer periods of time in their vehicles. Driver fatigue is a major issue among road hauliers as it amplifies the risk of accidents. Despite European legislation regulating driving times, many drivers don’t return home for months (Interview BRU-3, 2025; Vitols and Voss, 2021). From this view, spatial-temporal compressions regarding truck drivers involves the lengthening of the working day to earn sufficient wages to survive and the increase in spatial distances, especially cross-border work within the EU- both of which are embodied in their labour power (Interview BRU-3, 2025; RTDD, 2023). The capitalist state at various scales (EU, national and municipal) has played a key role in shaping and reconfiguring highways and the road freight industry, including the labour power of road hauliers along spatial and temporal lines.

#### Capitalist state scales

This brings us to the third HGM analytic, the scalarity of the capitalist state. The capitalist state is a geographically and historically specific social relation that plays a crucial role in reproducing capitalist social relations embodied in highways and truck drivers’ labour power by addressing barriers to cost efficient and rapid movement of freight, such as traffic congestion and truck driver shortages. States do so by engaging in spatio-temporal restructurings aimed an annihilating space through time at various scales (Brenner, 2004; Chua, 2022). State scales shift and change as they mediate crises in capital accumulation by attempting to resolve the internal contradictions of capital accumulation comprised of a complex and ever-changing arrangement of moving parts at various scales of intervention (Brenner, 1997; Soederberg, 2021). Viewed as an arrangement of moving parts at various scales of intervention, each with its own set of nuances and tensions (Brenner, 2004), these multi-scalar mediations are incomplete, contested and generative of further contradictions and conflicts. Through varied legal and monetary powers, these state scales have played pivotal roles in forging time-space compressions to mediate contradictions internal to capitalism (Brenner et al., 2010). At the national scale, for instance, the German state deals with congestion on the A100 through a series of spatial extensions over the past 65 years (Escher, 2020). At the European scale, the EU has provided regulatory frameworks that encouraged faster delivery times at the lowest cost possible as it continually expanded the internal market.

Deploying the three analytics to connect capitalist state scales and truck drivers’ labour power, I now turn to a concrete analysis of the root causes, contradictions and conflicts underpinning the controversial 16th extension of the A100. This enables me to analyze how the continued prioritization of highway construction to move urban freight comes at the expense of social justice.

## Berlin’s A100 and the remaking of city logistics in global capitalism

### The making of the car-friendly city: 1950s–1960s

The A100 was perceived by urban planners and the Allied Powers as a vital component of modernizing West Berlin’s transport infrastructure to reduce inner-city congestion and offer an alternative route for cross-city circulation of people and goods (Escher, 2020). This modernization plan could be seen as a state-led attempt at remaking city logistics centred on creating a car- and truck-friendly city (*Autogerechtstadt*) to facilitate quicker and more efficient circulation of goods (Bernhardt, 2020). The A100 played a pivotal role in the restructuring of West Berlin’s economy. Its first segment opened in 1958 and connected the Kurfürstendamm to Hohenzollerndamm, a relatively affluent area in the district of Charlottenburg and key commercial hub based around the Kurfürstendamm. The highway was also in near proximity of some key manufacturing sites such as Siemenstadt (Zimm, 1961).

The erection of the Berlin Wall in 1961 imposed limits to the spatial expansion and thus city logistics of West Berlin in at least two ways. First, separated from its hinterland, West Berlin’s urban development proceeded with no or little suburbanization (Hesse, 2008). Second, the physical separation between West Berlin and the rest of West Germany meant that access routes were limited leading to the city’s heavy reliance on air and truck transportation (Kalendar, 2012). To support economic growth as well as to showcase Western freedom and the superiority of capitalism in West Berlin (Zimm, 1988), the West German state, with financial support from Allied Powers (Whyte and Frisby, 2012), financed and constructed six segments of the A100 culminating in 12.5 km from 1958 to 1969 extending the A100 from Funkturm in the district of Charlottenburg to Westrasse in the district of Schöneberg.^1^

### Inter-scalar space-time compressions: 1970s–1980s

Between 1976 and 1987, the West German state extended the A100 a further five times, adding a total of 15.7 km to Berlin’s intercity highway. Applying my HGM framing, I reveal how these highway extensions were an integral feature of at least two spatio-temporal restructurings involving, first, the expansion of the European Economic Community (EEC) to include cheaper labour from southern European countries; and, second, the centralization of the regulatory power over the road freight sector from national state scales to the European state scale (Brenner, 1997, 2004).

The post-war boom for West Germany started to slow by the 1970s, leading to lower growth rates across Western Europe and a steep increase in the price of crude oil that would hit automobile owners and the trucking sector particularly hard in West Berlin (Kalendar, 2012). In 1973 the EEC sought to counter stagnant growth, steep inflation and rising unemployment by expanding its membership to include southern European countries while ensuring regulations offered flexibility in hiring and firing rules to make labour costs cheaper and thus EEC goods more competitive on the world market (Cafruny and Ryner, 2003). The Schengen Agreement (1985) and Single European Act (1985) established the foundation for deeper market integration, which made cross-border subcontracting possible (Erne et al., 2024). Subcontracting road freight describes a situation in which large logistics companies (main contractor) hire smaller and medium-sized road freight companies (subcontractor) (Borelli, 2022). Subcontracting in the European logistics sector often involves social and wage dumping, whereby a large contractor in a Western European country such as Germany takes advantage of the relatively lower wages, taxes and benefits in a CEE country, such as Poland (De Smedt and Wispelaere, 2020).

These spatial restructurings of the EEC were accompanied by temporal compressions of the road freight sector. The latter was seen as prime importance to the completion of the internal EEC market given the role it played in facilitating trade, representing more than 7% of the EEC’s gross domestic product (Commission of the European Communities, 1985: 29). The significance of road freight sector in the expanded EEC led to a shift in power over this sector from national states to the European state scale (Knill and Lehmkuhl, 2002). The rationale for this was to ensure uniformity to the flow of goods across Member States to facilitate faster speed of circulation of commodities (Erne et al., 2024). More intensive road freight circulation also required highways that could facilitate faster turnover times from the point of production to the point of consumption (Commission of the European Communities, 1985; Harvey, 2001). While railways began to lose their dominance in Germany as the main freight mover in the previous two decades, road freight overtook rail as the main freight carrier in the 1980s as road transport companies could offer more flexible, cost effective, and faster delivery of goods than rails (Hilal, 2008).

In 1973, the German state initiated its first Federal Transport Infrastructure Plan (hereafter: BVWP), which was an important, legally binding roadmap for planning and financing mid- to long-term investments in highways, rails and waterways in the country. The 1973 report observed that highway traffic in Berlin-Templehof, where the A100 was located, was growing steadily, leading to delays and possible traffic jams (Deutscher Bundestag, 1973: 96). This laid the groundwork for the five extensions to the A100 from 1976 to 1987 to ensure efficient circulation of urban freight by alleviating traffic congestion in West Berlin. The additional phases of the A100, for instance, facilitated faster movement of trucks between Westhafen GVZ and other industrial zones in West Berlin and West Germany (Escher, 2020). Not everyone in West Berlin agreed with these construction phases, however. As early as 1970, citizen groups and environmental organizations were protesting the plan to lengthen the A100, arguing that more highways were not a cure-all for traffic congestion. The BI Westtangente was one of the most formidable groups challenging, among other issues, the extensions of the A100 (Berliner Morgenpost, 1970; Engler, 2020; Interview B-14).

### Reunified Berlin and EU integration: 1990–2003

Between German reunification in 1990 and 2004, the federal government extended the A100 a further three times, lengthening the intercity highway by 7.2 km eastwards from Sachsendamm in the district of Schöneberg to Grenzallee in the district of Neukölln borough, where it merges with the A113 (see Figure 2). The latter motorway serves as a key connector of the capital city to the Berlin Brandenburg Airport (BER), which began construction in 2006 (see Figure 2). The A113 connects Berlin to Grossbeeren IFC, which would become a leading logistics hub, ranking within the top five in Europe (EUROPLATFORMS, 2015). In this section, I explain how these three construction phases of A100 were entangled in the spatio-temporal compressions of global capital accumulation. First, I elaborate the wider liberalization processes involved in the integration of the European market and, by extension, significant deregulation of the road freight sector by specifically focusing on truck drivers’ labour power. Second, I emphasize how these compressions connected to the lengthening of the A100 and the remaking of Berlin’s city logistics through the establishment of GVZs outside of the capital (Hesse, 2008).

Facing low growth rates throughout the 1990s, the European state scale complemented the expansion of its markets by compressing the circulation time of goods and services across space (Cowen, 2014; Harvey, 2001). This restructuring was primarily accomplished with the signing of the Treaty of the European Union in 1992 – or, what is more popularly known as the Maastricht Treaty. Among other things, the Maastricht Treaty ensured the *four freedoms*, that is, free flow of capital, labour, goods and services across the internal market by reducing barriers to entry and thus opening these sectors to competition. These series of liberalizations not only ensuring that market-friendly EU law took precedence over more restrictive national laws (Ojala, 2020) but also enshrined the prioritization of economic interests over social concerns (Bieler and Morton, 2018). While not a direct feature of the Maastricht Treaty, a well-functioning internal market required a cost efficient and speedy flow of goods across and within national borders (Plehwe, 1998). The combination of the prioritization of economic over social concerns, on the one hand, and accelerated circulation of goods in the EU, on the other hand, would converge in the deregulation of the road freight sector, especially the regulations governing transport workers (Hilal, 2008).

In the wake of the completion of the EU Single Market, the EU passed legislation that would enable accelerated and cost-effective movement of goods across national borders by further deregulating the road freight sector (Erne et al., 2024). By 1998, road freight transport within the EU was completely deregulated, allowing unrestricted market competition without any quotas or limitations (Hilal, 2008; Interview BRU-3, 2025; Interview BRU-5, 2025). These EU-led space-time compressions facilitated an increased subcontracting activities in the road freight sector and significantly affected road cabotage. According to the EU, cabotage describes the transport of goods between two locations within the same country by a haulier registered in another EU Member State (European Commission, 2009; Tvedt, 2024). In 1993, the EU deregulated cabotage rules (Reg. 3118/93) to make it easier for non-EU hauliers (largely in CEE countries) to operate within EU Member States under specific conditions (Broughton et al., 2024). Western European logistics companies, for example, DHL Freight, DB Schenker, Kuehne + Nagel, gradually subcontracted to CEE transporters – particularly from Poland, Romania and Bulgaria (De Smedt and Wispelaere, 2020).

During this deregulatory environment, letterbox companies emerged in CEE countries to evade transport workers’ rights, including cabotage rules, and exploit wage differentials across the EU. Letterbox companies are legal entities set up by businesses to benefit from a regulatory framework in a jurisdiction in which they have little or no material operations and allow for *regime shopping* for lower taxes, wages, labour standards, social contributions, and so forth (McGauran, 2016; RTDD, 2020). As I discuss in the next section, these subcontracting practices became more pronounced in with eastward enlargement of the EU in 2004 and 2007 whilst road freight transport increased (Borelli, 2022; Eurostat, 2009).

These state-led restructurings also shaped the remaking of logistics in Berlin- a city that was undergoing demographic and economic changes in its expanded reunified urban space with a large hinterland, including the state of Brandenburg (see while areas in Figure 2). The swift increase in Berlin’s population coupled with the meteoritic rise in e-commerce in the 1990s (Germany is one of the EU’s largest markets) led to an increased number of trucks to deliver the growing demand of goods (Aoyama and Schwarz, 2013). Indeed, by 2000, road freight transport accounted for around 70% of inland transportation of goods (Eurostat, 2008). To create a more efficient and controlled flow of urban freight – particularly to support the increasingly busy inner-city Westhafen GVZ (Röber, 1991) – the Berlin Senate and the state of Brandenburg collaborated to subsidize the relocation of freight and warehousing services from the city to suburban areas.

A key criterion for site selection was the availability of large industrial land parcels with room for expansion. Equally important was access to fast and reliable transportation infrastructure, including motorways and federal highways such as the A100. Crucially, the absence of traffic disruptions was a leading factor in selecting locations for logistics hubs outside Berlin (Klauenberg et al., 2020). The suburban spaces in Brandenburg met these requirements and more, becoming strategic sites for international freight centres or GVZs (Hesse, 2008; Hesse and Rodrigue, 2004; Interview B-7, 2025). Although branded as situated in Berlin to attract potential clients by signalling the proximity to the German capital (Interview B-7, 2025; Interview B-16, 2025), the GVZs were located in the state of Brandenburg, which surrounds the city-state (Figure 2).

The establishment of three GVZs in strategic locations around Berlin to its West (Wustermark GVZ), to its East (Freienbrink GVZ), and one of the largest GVZs in Europe to the South of Berlin (Grossbeeren GVZ) would transform reshape city logistics in Berlin by reconfiguring the distribution of urban freight to flow from the GVZs to the inner-city consumption sites, that is, households and businesses (Hesse, 2008; Klauenberg et al., 2020). Many trucks originating from Grossbeeren GVZ, Freienbrink GVZ and Wustermark GVZ, rely on the A100 to reach urban delivery zones (Interview B-16, 2025). Coinciding with the emergence of GVZs, the importance of efficient flows of traffic along the A100 was first acknowledged in the 1992 Federal Transportation Infrastructure Plan included the 16th extension of the A100 from Dreieck Neukölln to Treptower Park to streamline distribution to various urban districts in Berlin (Bundesministerium für Verkehr, 1992; see Figure 1).

As these GVZs grew in the late 1990s, they placed increasing pressure on the already congested A100. In response, the federal state acted to alleviate these bottlenecks to ensure the faster flow of goods into and across the German capital (Klauenberg et al., 2020). In its 2003 Federal Transport Infrastructure Plan, the German state took the bold move by categorizing the 16th extension of the A100 as *necessary* (Vordringlicher Bedarf) signalling the prioritization of the extension for funding and construction due to the economic significance of the A100 to Berlin and Germany (Bundesministerium für Verkehr, Bau- und Wohnungswesen, 2003: 97).

### Eastward time-space compressions: 2004–2012

In 2004, the EU undertook the biggest enlargement in its history by expanding its markets eastwards, where, among other factors, labour was relatively cheaper. In 2007, the EU with strong German support widened it membership base further to include Bulgaria and Romania (Erne et al., 2024). These spatial expansions facilitated nearshoring of production – a trend whereby companies in Western European member states relocated business operations to the newly joined Eastern European Member States to take advantage of lower labour costs (Bieler, 2002). A larger European market also meant that truck freight companies would come under pressure to ensure faster and cost-effective turnover times across bigger spaces of the internal market. In the deregulated environment of the road freight sector coupled with access to cheaper labour markets, cabotage became an increasingly important feature of the trucking landscape, especially in the GVZs surrounding Berlin. It is worth emphasizing here that the German state predicted a rise of 84% in long-distance road haulage from 2004 to 2025 – over four times the increase in passenger traffic (Federal Government (of Germany), 2017: 11).

Following deregulation in 1998 – in which road hauliers were allowed up to three cabotage operations within 7 days following an incoming international carriage – its prevalence in Germany increased from 1.9% in 2004 to 7.2% in 2017 (European Commission, 2009). Over 50% of cabotage driving in Germany has been carried out by Poles, with Romania as the second largest in terms of transportation labour. As I mentioned earlier, sub-contracting has become a central feature of the trucking sector, including cabotage activities. This practice is cheaper than directly hiring drivers in Germany and thus paying German wages, as the contractor does not have to pay benefits, taxes or full-time salaries to contractors (Tvedt, 2024). Some of the main contractors of cabotage are large logistics companies such as DB Schenker, Kuehne + Nagel, DHL, who have constructed complex subcontractor chains and who operate out of the GVZs surrounding Berlin, especially the Grossbeeren GVZ (Interview B-10, 2025; Sternberg et al. 2020).

As I noted earlier, in the wake of the EU enlargements in 2004 and 2007, letterbox companies became a major policy issue, particularly prevalent in the road freight sector. Combined with the EU Posting of Workers Directive (1996, revised in 2018 see European Parliament, 2018), letterbox companies are permitted to send employees temporarily to another EU country to provide services (Šimurková and Poliak, 2019). For example, a letterbox company in Poland can send truckdrivers to deliver goods in Germany, but since the company is legally Polish, it pays the drivers according to Polish wage levels and social insurance contributions (McGauran, 2016). Between 2004 and 2017, for example, the transport work carried out drivers posted by Polish companies in Germany increased fivefold (De Smedt and Wispelaere, 2020).

Immediately after the 2007 enlargement, the EU was impacted by the global financial crisis that elicited restructuring at all state scales to overcome barriers to capital accumulation (Bieler and Morton, 2018). These measures placed social concerns such as those in the road freight sector in a relatively subordinate position, resulting in the entrenchment of inequality and social exclusion (Erne et al., 2024). In 2009, the German state responded to its negative growth rates by launching a €50 billion stimulus package (*Konjunkturpacket II*), which earmarked €17.3 billion for core infrastructure projects, including highway builds (Bieler and Morton, 2018).

After the two enlargements in 2004 and 2007, Germany repositioned itself spatially as the Gateway to the East due to its central location. This further consolidated its importance as the logistics hub of the EU (Bundesministerium für Verkehr, Bau- und Wohnungswesen, 2008). Berlin gained significance as a major logistical bridge between Western and Eastern Europe. This, along with access to cheaper labour through cabotage and letterbox companies, led to a significant expansion in cross-border road transport (Eurostat, 2009). The GVZs near Berlin, such as Grossbeeren, became transshipment points, storage hubs, and logistics hubs for goods entering or exiting Germany to the CEE Member States (Interview B-16, 2025; Klauenberg et al., 2020). The European Regional Development Fund (EDRF) played a significant role in financing the expansion of the GVZs in the Berlin-Brandenburg region after EU enlargements, particularly Grossbeeren, which became one of the largest freight villages in Europe in 2005 (Economic Development Agency Brandenburg, 2019).

Since most of the freight flowing in and out of the GVZs were moved by road hauliers, key road connections required upgrading, including an extension to the A100, to address congestion. Eager to receive financing from the stimulus package, business groups associations (e.g. Industrie- und Handelskammer Berlin, Unternehmensverbände Berlin-Brandenburg), including firms tied to the GVZs, argued that without the 16th extension, traffic congestion would hamper Berlin’s competitiveness (Investitionsbank Berlin, 2024). Building the 16th extension was thus seen as a necessary move to facilitate faster flow of road freight into and across Berlin, along the highly congested A100. According to its supporters, the 16th extension would allow drivers from the districts of east Berlin to enjoy faster access to the city, regional and long-distance road networks (IHK, 2024b). After a brief public consultation in 2009, the Federal Ministry of Transport approved funding for the A100 extension in 2011 (Bundesministerium für Verkehr und digitale Infrastruktur, 2016).

### The 16th and final highway extension? 2013–2025

Building on my previous analysis of how past construction phases of the A100 have been intertwined with the continual attempts by the capitalist state at various scales of intervention to annihilate space through time in global capitalism, in this section I explore the temporal period from the start of construction of the 16th extension in 2013 to its scheduled completion in 2025. I bring to the fore three developments arising from state-led compressions aimed at facilitating faster and more cost-effective circulation of commodities (road freight and its drivers) across Berlin city logistics. First, I explore the conflicts surrounding Berlin’s new highway extension aimed at decreasing the turnover time for urban freight. Second, I examine attempts by the EU to manage the contradictions emerging from decades of deregulated road freight sector, for example, an acute shortage of truck drivers, through the introduction of the Mobility Package in 2020. Third, I scrutinize the proposed 17th extension to the A100 and the newly formed national government agency, Autobahn GmbH, as examples of the incomplete nature of spatio-temporal compressions. While each section highlights the incomplete, conflictual and contradictory features of state-led restructurings, these three attributes are fluid.

#### Conflict-led compressions

On 8 May 2013, the Federal Transport Minister Peter Ramsauer (Christian Social Union or CSU) and Berlin’s Senator for Transport Michael Müller (Social Democratic Party or SPD) conducted a groundbreaking ceremony for the 16th construction phase. The lengthening the intercity A100 reflected a spatio-temporal compression carried out by the German state to ensure that urban freight can flow quicker into and across Berlin. At the same time, this reconfiguration of city logistics entailed destruction of affordable housing dwellings, over 12 hectors of green space and numerous nightlife institutions belonging to Berlin’s longstanding iconic club culture (Abgeordnetenhaus Berlin, 2023a). The 16th extension was met with fierce resistance from a variety of groups and organizations ranging from national organizations (Friends of the Earth Germany, Extinction Rebellion Germany, Letzte Generation) to local residence groups (Bündnis A100 Stoppen, Berlin Club Commission).

Opposition also came from various state scales, revealing internal divisions within the capitalist state. The Berlin Senate (Red-Green coalition), which was in power at the time, was divided over the 16th construction phase, with the SPD supporting the construction, with the Greens (Bündnis 90/Die Grünen) and the Left (die Linke) opposing the extension on environmental and social concerns (Interview B-9, 2025; Senatsverwaltung für Mobilität, Verkehr, Klimaschutz und Umwelt, 2021). A similar division was reflected in the federal state, with left-leaning parties (Greens and die Linke) in the federal parliament (Bundestag) opposed to both the 16th and proposed 17th extension (Deutscher Bundestag, 2022). Through question periods and open debates about the further construction of the A100 in the Bundestag, the left-leaning parties publicly exposed the class nature of the highway build. It was argued, for instance, by the Left (die Linke) that the highest cost of the extension will be paid by lower-income groups, who will live next to the highway and thus be directly exposed to noise and air pollution (Deutscher Bundestag, 2022; Interview B-14, 2025).

The German state defended the highway build by emphasizing the economic importance of highways in circulating the increasing volume of urban freight more efficiently. In its most recent Federal Transport Infrastructure Plan (2016–2030), the Federal Ministry for Digital and Transport estimates that road freight transport will increase by 38% in 2030 compared to only a 12.2 increase in passenger vehicles over the same period (Bundesministerium für Verkehr und digitale Infrastruktur, 2016). To facilitate this growth in road freight, and, by extension, global supply chains, the German state has allocated almost half of its total budget for transport infrastructure (€270 billion) to roads, and particularly highways such as the A100 (€132.8 billion; Bundesministerium für Verkehr und digitale Infrastruktur, 2016). Moreover, the German state justifies the cost of the 16th extension (and eventually the 17th) as necessary to modernize transport networks to ensure quicker circulation time as these are the basis for ‘growth, prosperity and employment’ (Bundesministerium für Verkehr und digitale Infrastruktur, 2016: I).

The Berlin Chamber of Commerce (IHK) has emphasized the importance of the A100’s 16th extension, citing its role in maintaining the integrity of global supply chains and supporting broader economic growth (Investitionsbank Berlin, 2024; Interview B-16, 2025). With approximately 230,000 vehicles using the A100 each day – many of them involved in urban freight transport (Senatsverwaltung für Mobilität, Verkehr, Klimaschutz und Umwelt, 2024) – both the 16th construction phase and, as discussed below, the proposed 17th extension are viewed by Berlin-based business interests as essential for ensuring the fast movement of goods across the city and keeping transportation costs low (IHK, 2024a, 2024b; Interview B-16, 2025). While the GVZs (freight villages) surrounding Berlin provide intermodal options, including rail, waterways, and air, road freight remains the dominant mode of transport for these distribution and warehousing hubs (Klauenberg et al., 2020). For logistics companies such as DHL, DB Schenker, and Amazon, which operate out of these intermodal freight centres (GVZs), the A100 serves as a key urban artery enabling rapid access to and through the city (Berlin Business Location Centre, 2024). In practice, trucks rely on the A100 to connect with other major highways, including the A10, A111, A113, and A115 (see Figure 2).

#### Contradictory compressions

As my analysis has made clear, space-time compressions involve more than highway extensions. Depressing circulation costs of freight does not solely depend on congestion-free highways, but also decreasing wages paid to truck drivers and, in so doing, coercing them to increase their labour time to cover longer distances in shorter periods of time (Vitols and Voss, 2021). Since the EU-led deregulation of the trucking sector in 1998, new logistical frictions have emerged ultimately impeding circulation time and destabilizing commodity flows. Some key issues facing the sector include a high vacancy rate due to poor working conditions and growing levels of precarity among truck drivers (RTDD, 2023). Industry associations and think tanks estimate that there currently is a shortage of anywhere from 380,000 to 600,000 drivers in Europe, which is indeed worrying in the face of growing road freight in Germany and indeed across the EU (Interview BRU-2, 2025; Interview BRU-3, 2025; Interview BRU-4, 2025; Interview BRU-5, 2025). A 2021 report by the European Labour Authority, for instance, identifies heavy truck driving as a top shortage occupation in the EU (McGrath, 2021).

A major contributing factor to these labour shortages has been poor working conditions and low way – both of which have been part the growing competition of cheaper sub-contractors in the road freight sector in light of EU deregulation. Most of these sub-contracting firms are quite small. For instance, 89% of truck operators employing have less than 10 employees – and the majority of are based in CEE Member States, particularly Lithuania and Poland (Interview BRU-3, 2025; IRU, 2024). Many of the larger logistic companies (Kühne + Nagel, DB Schenker, DHL Freight, Amazon Logistics) operating out of the GVZs surrounding Berlin have shifted to an ‘asset-light’ model meaning they own little or no fleet but instead rely heavily on subcontractors (Borelli, 2022; Interview B-10, 2025; Interview BRU-3, 2025). This, in turn, makes it very difficult to discern where road freight company begins and the logistic companies and their role in coordinating, planning and optimizing freight movement, warehousing and delivery end (De Smedt and Wispelaere, 2020).

Since 2004, with the eastward expansion of the EU, many subcontracting logistics companies often from Western European Member States (e.g. Germany, Austria, France, and the Netherlands) have set up letterbox companies in CEE Member States to take advantage of lower labour costs, low social protection standards, low tax regimes, and, more generally, reduced regulation (European Transport Workers’ Federation, 2023a). These letterbox companies dispatch drivers across the EU, especially Germany, the Netherlands, and France. Although these drivers posted in Germany, for example, they are paid according to the wage structure and social benefits in the CEE Member State where their subcontractor is based. Poland is a major player with almost 20% of total EU road transport performed by vehicles registered in that country (McGauran, 2016). To overcome the problem of labour shortage and to further reduce transport costs and thus expand their profit margins, many of these subcontracting firms based in CEE Member States have employed third-country nationals (e.g. Central Asia, Philippines, Zimbabwe, and the Ukraine) further evading social protections and labour regulations (RTDD, 2023).

In 2017, the European Commission introduced the Mobility Package to address these concerns facing the European road transport sector by focusing on fair competition and drivers’ working conditions, often undermined by letterbox companies (European Commission, 2020; Interviews BRU-4, 2025). The Mobility Package represented one of the most contentious pieces of legislation? In the EU transport sector, leading to several years of negotiations. Western European countries including Germany, for instance, sought to introduce stronger regulations to prevent wage undercutting and improving the working conditions for truck drivers – often linked to subcontracting – whilst protecting local markets. This was met with fierce opposition from CEE Member States, who sought to protect their competitive edge (European Transport Workers’ Federation, 2023a). In 2020, the Member States agreed to commit to the ratified Mobility Package, which offered a comprehensive framework for regulations (legally-binding) enforceable across all EU Member States. Key legal instruments include driver and rest time rules, cabotage and posting rules, ensuring that international freight drivers receive fair wages and working conditions (European Commission, 2020; Interview BRU-5, 2025; Interview BRU-2).

While the reforms introduced by the Mobility Package have been widely lauded across by various stakeholders of the freight transport industry, it has also come under heavy criticism by many national and European trade unions (Ver.di, European Transport Workers’ Federation) and social justice organization (Faire Mobilität, RTDD). According to these critics, the main weakness of the Mobility Package is its lack of compliance by Member States (Interview BRU-3, 2025). Moreover, much of the responsibility to follow the Mobility Package regulations end up falling on the shoulders of individual drivers, not the freight and logistics companies who are employing them (European Transport Workers’ Federation, 2023a; Interview BRU-3, 2025). The immediate consequences of the inability to reinforce the Mobility Package has led to the continued precarity and poor working conditions facing many truck drivers, with the worst employment conditions experienced by the growing number third country nations (Interview B-8, 2025; Interview B-10, 2025; RTDD, 2023). An informant from a European road hauliers’ employers’ association referred to truck drivers as the *weakest link* in global supply chains (Interview BRU-2). This is particularly true of non-EU countries, who are often forced to sign contracts in languages they cannot read and are compelled to rest, sleep, east and live in their vehicles for months on a continual basis. For these long hours, they are remunerated with extremely low monthly wages around €100–€600 per month, with unpaid wages a common issue among drivers (RTDD, 2020).

This structural violence of space-time restructurings experienced by road hauliers has not been met with acquiescence. In 2023, a widely publicized strike took place in Gräfenhausen, Germany where over 60 road hauliers from third countries employed by a Polish logistics company (Mazur Group) protested to draw attention to their unpaid wages and poor working conditions (Benedetti, 2023). The strike exposed the indirect involvement through subcontracting of major multinational corporations, including IKEA, Volkswagen and DHL (European Transport Workers’ Federation, 2023b). The successful resolution of this strike, which involved the remuneration of overdue wages, was followed by another strike and hunger protest involving over 130 drivers also from non-EU countries to demonstrate the ongoing plight of workers. Mazur Group agreed to pay the outstanding wages and dropped legal actions against the strikers (European Transport Workers’ Federation and International Transport Workers’ Federation, 2023).

The combination of complex subcontracting chains of logistics with the mixed efficacy of the Mobility Package has resulted in the continued issue of the widening vacancy gap of professional drivers in the EU (European Transport Workers’ Federation, 2023a). This issue has been met with further spatio-temporal compressions. With around 5% of its workforce under 25 years of age (with only 2.6% of road hauliers in Germany are under 25), the EU updated its drivers licence directive in March 2025 to lower the minimum age for obtaining a truck driving licence from 21 to 18 thereby (Broughton et al., 2024). Moreover, Member States may permit 17-year-olds to drive trucks under the supervision of an experienced, licensed driver (Interview BRU-4, 2025). Alongside this temporal restructuring regarding the modification of age requirements, the EU is updating its immigration rules to permit the employment of qualified drivers from non-EU countries. Spatially expanding its labour pool through the inclusion of third-country nationals and lowering the minimum driving age, the EU has sought to address the expected shortfall of 745,000 truck drivers by 2028 (IRU, 2023). Despite these initiatives, however, it is unclear how existing structural conditions of the road hauliers’ labour markets such as poor working conditions, including driver fatigue and its major risks factors, and low wages through subcontracting channels will fill the significant shortage of truck drivers in the EU. As a recent posting by the European Transport Workers’ Federation (2025) observed, ‘Gräfenhausen is not an isolated case; exploiting third-country nationals is a growing business model that risks corrupting a sector regulated by one of the best articulated EU legal frames but battered by poor enforcement’.

As the EU state scale is attempting to resolve conflicts and contradictions in the road freight sector, further spatio-temporal restructurings were being debated in Berlin.

#### Incomplete compressions

As the final segment of the 16th extension of the A100 was well under construction, various political parties at the federal state scale (CDU and Free Democratic Party, FDP) and business interests (e.g. German Transport Association, BGL and Federation of German Industries) began supporting the construction of a 17th extension. This highly controversial extension would run 4.1 km from Treptower Park to Storkower Strasse (see Figure 1).

The 17th construction phase would cost taxpayers over €1.1 billion thereby easily usurping the 16th extension status as the most expensive highway in Germany’s history (Interview B-14, 2025; RBB24, 2025). The groundwork for the idea of the 17th extension was acknowledged in the 2016 Federal Transport Infrastructure Plan but has not yet been given immediate priority planning (Bundesministerium für Verkehr und digitale Infrastruktur, 2016). Its supporters stress the importance of this extension to more efficient transportation in the city including freight mobility. By absorbing through-traffic (particularly trucks), the 17th extension is argued to reduce congestion, emissions and noise in neighbourhoods and better connect eastern districts of Berlin (such as Friedrichshain and Lichtenberg). The A100 is also seen as an indispensable addition to the transport network linking the city to BER Airport, which was completed in 2020 (IHK, 2024b). While the SPD in the Berlin Senate was committed to the 16th extension, it remained in solidary with its left-leaning Green-Red coalition (2016–2021) in opposing the 17th extension of the A100 as it clashes national and city-state’s climate and sustainability goals (Abgeordnetenhaus Berlin, 2023b). The Wegner Senate, a Black-Red (CDU-SDP) coalition which came to power in 2023 is split in its support for the 17th extension, with some Senators from the SDP, Greens and the Left opposing the project (Interview B-12, 2025). Although the final decision to reject a federal highway build belongs to the German state – typically, the Ministry for Digital and Transport – the Berlin Senate can influence this decision through political pressure, legal challenges, environmental impact assessment and planning, and so forth (Engler, 2020; Karapin, 2007). Some Berlin Senators in allegiance with grassroots movements involved in the rejection of the 16th extension are seeking to deploy these instruments of power to stop the approval process, which is slated to begin in 2027 (Interview B-5, 2024; The Berliner, 2023).

Against the backdrop of these debates, the German state advanced internal reorganizations to help deliver more controlled and efficient logistics spaces in the country. To this end, the federal state it created a centralized agency, the Autobahn GmbH des Bundes (Highway Ltd.) that came into force on 1 January 2021. While the Ministry for Transportation and Digital would still shape general policy, especially through the publication of its Federal Transport Infrastructure Plans (BVWP), the mandate of the Autobahn GmbH is to manage, plan, build, operate and maintain the entire federal motorway, including in city-states such as Berlin (autobahn.de/ueber-uns/was-wir-tun, Interview B-15, 2025). Indeed, the Autobahn GmbH is responsible for the implementation of the 17th extension of the A100. The Autobahn GmbH has been criticized for its lack of transparency regarding the tendering process involving companies hired to plan and design services for the 17th extension as well as the agency’s poor communication with the public about it projects and decision-making (Abgeordnetenhaus Berlin, 2022). In many ways, the Autobahn GmbH serves to obscure oversight and close off political discussions about highway builds, which was confirmed by my informants in the Berlin Senate and Bundestag (Interview B-12, 2025; Interview B-14, 2025).

Notwithstanding the centralized and technical governance of the proposed 17th extension by the Autobahn GmbH and the continued structural violence of road hauliers, these state-led restructurings aimed at accelerating the circulations of commodities into and across Berlin are not only being continually contested and challenged in the name of social justice. They also reveal incomplete, conflict-ridden and contradictory nature of spatio-temporal compressions.

## Conclusion

Inspired by the commitment of radical geography to *defetishizing* our world (Harvey, 1990), I sought to contribute to studies on highway infrastructure by exploring the capitalist nature of a largely neglected yet highly contentious intercity highway A100. In so doing, I asked how we might explain the historical tensions, tendencies and trajectories of the 16th extension of the intercity highway in the socio-spatial and temporal dynamics of global capitalism. A HGM lens facilitated an understanding of highways as social relations, opening conceptual pathways to examine the connections between road freight drivers and the state scales in capital accumulation. I argued that the past, present, and proposed extensions of the intercity A100 are emmeshed with crises, contradictions, and class dynamics of global capitalism. Following the A100 from its opening in 1958 to its proposed completion in 2025, I suggested that the capitalist state at various scales of intervention has played crucial yet contradictory, incomplete and contested roles in forging what Harvey (1999) describes as time-space compressions. I reveal how different scales of the capitalist state have acted to reconfigure temporal and spatial relations to provide faster and cheaper circulation of commodities. These overlapping reconfigurations aimed at annihilating space through time – ranging from the prolonged construction of the A100 to successive expansions of the European internal market with fewer regulatory restrictions on the movement of goods and labour – provided the framework through which road hauliers could be compelled to work longer hours for less pay, thereby covering greater distances across space.

Providing a radical highway geography approach to the A100 yields several key insights. First, the intercity highway, as a special type of fixed capital, has become increasingly vital to capital accumulation and value production as Berlin became more deeply integrated into the internal European market. This trajectory intensified since reunification in 1990, with state-led efforts to deepen Berlin’s position as a major logistics hub in the EU through the development of its GVZs. Second, the requirement of capital for faster and cheaper circulating goods across space through truck freight consistently took priority over concerns road hauliers’ working conditions and remuneration. The major intervention of the Mobility Package in 2020 sought to address rampant social and wage dumping taking place in the freight industry’s subcontracting chains, but only when motivated by potential limitations to flow of global supply chains were under threat, including acute driver shortage. Unlike other observations on these issues, my HGM analysis uncovers the underlying socio-spatial and temporal restructurings of the capitalist state across various scales of intervention, as well as the capitalist and class dynamics driving these transformations and their inherent paradoxes.

A third insight has been increasingly fragmented landscape of the road freight industry, with many companies registering under 10 employees, and its obscure connection to larger logistic companies through subcontracting and letterbox companies has been the result of the EU-led liberalization and deregulation agenda the broadly began with the Single European Act in 1986 and accelerated with the expansion of the internal European market in the 2000s. Fourth, and relatedly, my analysis builds on Harvey’s (1999) insight that fixed capital – such as the A100, a special type of fixed capital – is not merely a physical remnant of capital accumulation. Truck drivers who traverse highways like the A100 also embody the spatio-temporal compressions that are central to capital accumulation.

Ultimately, a radical geographic lens opens analytical pathways that move beyond viewing highways as static objects, enabling a connection of ‘distant strangers whose contributions to their lives were invisible, unnoticed, and largely unappreciated’. It reveals how these actors have both shaped – and continue to be shaped by – the socio-spatial and temporal dynamics of capital accumulation (Cook, 2004: 642). Such an approach offers a valuable step toward exposing the class dynamics and contradictions inherent in capitalism, while challenging what has become the default response of capitalist states to disruptions in urban freight flows: building more highways. This tendency is underscored by a troubling trend – roads remain the highest category of infrastructure investment across the G20 countries (Global Infrastructure Hub, 2023).

By getting ‘behind the veil, the fetishism of’ highways (Harvey, 1990: 422), it becomes possible to reconnect fragmented, multiple, and often contradictory struggles for justice – ranging from third-country road hauliers protesting wage theft and demanding better working conditions in Gräfenhausen to ongoing resistance movements in Berlin opposing the 17th construction phase of the A100 on environmental grounds. Reconnecting highways to city logistics in global capitalism provides fertile ground to contest the exploitative and destructive nature involved in the state and capitalist led strategies to continually quicken and cheapen the spatial mobility of commodities whilst overcoming limits of immobile highway transportation.

Finally, thinking of the (defetishized) highway as a research site allows scholars to begin to see how seemingly contradictory justice movements – non-unionized third-country hauliers protesting wage theft, environmental activists opposing highway construction, and left-leaning factions of the state (Alliance 90/The Greens and The Left Party) – might, in fact, reinforce one another’s aims. As Clover (2016) has shown in their work on circulation struggles, progressive alliances – particularly with production struggles – are notoriously difficult to build, fractured by ideological, national, material and identity-based divisions. Yet in a world shaped by overlapping crises – social reproduction and the climate emergency – that are global in nature, these unexpected coalitions across class, cause, and community may offer the most effective resistance to the structural violence embedded in the temporal-spatial restructurings that enable the ever-accelerating movement of commodities across space – for, as Marx (1990) put it, the sole purpose and benefit of accumulation is accumulation itself.

## Notes

1. This extension also included the construction of the Ringbahn bridge in 1963, which joins the A115 (see Figure 1). A widening crack in the supporting structure of the Ringbahn bridge by the Funkturm was discovered in early 2025. The bridge was subsequently closed as it requires complete demolition and rebuilding. This construction will disrupt the flow of traffic, ‘as around 95,000 vehicles cross the three-land motorway bridge every day’ (Berlin Senate, 2025).

## References

[bibr1-0308518X251361632] Abgeordnetenhaus Berlin (2022) Schriftliche Anfrage- Abschluss A100 – wer ist wofür zustädig? 19. Wahlperiode. Drucksache 19/10 524. Berlin: Abgeordnetenhaus von Berlin.

[bibr2-0308518X251361632] Abgeordnetenhaus Berlin (2023a) A 100 stoppen und qualifiziert beenden! 19. Wahlperiode. Drucksache 19/1135. Berlin: Abgeordnetenhaus von Berlin.

[bibr3-0308518X251361632] Abgeordnetenhaus Berlin (2023b) Keine Verlängerung der A100- Planuungsstopp für den 17. 19. Wahlperiode. Drucksache 19/1139. Berlin: Abgeordnetenhaus von Berlin.

[bibr4-0308518X251361632] Angelo H Wachsmuth D (2015) Urbanizing urban political ecology: A critique of methodological cityism. International Journal of Urban and Regional Research 39(1): 16–27.

[bibr5-0308518X251361632] Aoyama Y Schwarz G (2013) From mail order to commerce: Competition, regulation, and the politics of nonstore retailing in Germany. Urban Geography 25(6): 503–527.

[bibr6-0308518X251361632] Benedetti G (2023) Dozens of truck drivers went on strike in Germany and won. Here’s how. Open Democracy, 6 June. Available at: https://www.opendemocracy.net/en/author/giulio-benedetti/ (accessed 4 March 2025).

[bibr7-0308518X251361632] Berlin Business Location Centre (2024) Freight Distribution Center (GVZ). Berlin: Business Location Centre. Available at: https://www.businesslocationcenter.de/en/infrastructure/freight-distribution-center-gvz (accessed 24 February 2025).

[bibr8-0308518X251361632] Berlin Senate (2025) Full closure of the A100 for years: Crack in the Ringbahn bridge. Available at: https://www.berlin.de/en/news/9547573-5559700-closure-a100-crack-in-the-ringbahn-bridg.en.html (accessed June 2025).

[bibr9-0308518X251361632] Berliner Morgenpost (1970) ‘Stadtautobahn nach Bucknow ist kein Allheilmittel für die Zukunft. Berliner Morgenpost, 20 October.

[bibr10-0308518X251361632] Bernhardt C (2020) The making of the ‘Stadtautobahn’ in Berlin after World War Two: A socio-histoire of power about urban automobile. The Journal of Transport History 31(3): 306–327.

[bibr11-0308518X251361632] Bieler A (2002) The struggle over EU enlargement: A historical materialist analysis of European integration. Journal of European Public Policy 9(4): 575–597.

[bibr12-0308518X251361632] Bieler A Morton AD (2018) Global Capitalism, Global War, Global Crisis. Cambridge: Cambridge University Press.

[bibr13-0308518X251361632] Bonacich E Wilson JB (2008) Getting the Goods: Ports, Labor, and the Logistics Revolution. Ithaca, NY: Cornell University Press.

[bibr14-0308518X251361632] Borelli S (2022) Subcontracting: Exploitation by Design, Tackling the Business Model for Social Dumping. Brussels: The Left in the European Parliament.

[bibr15-0308518X251361632] Brenner N (1997) State territorial restructuring and the production of spatial scale: Urban and regional planning in the Federal Republic of Germany, 1960-1990. Political Geography 16(4): 273–306.

[bibr16-0308518X251361632] Brenner N (2004) New State Spaces: Urban Governance and the Rescaling of Statehood. Oxford: Oxford University.

[bibr17-0308518X251361632] Brenner N Peck J Theodore N (2010) Variegated neoliberalization: Geographies, modalities, pathways. Global Networks 10(2): 182–222.

[bibr18-0308518X251361632] Broughton A Tanis J Brambilla M , et al (2024) Trends, Challenges, and Opportunities in the EU Transport Labour Market. Brussels: European Union.

[bibr19-0308518X251361632] Bundesministerium für Verkehr (1992) Der Bundesverkehrswegeplan 1992 – ein zukunftsorientiertes Infrastru-kturprogramm in einem integrierten Gesamtverkehrskonzept. Berlin: Bundesministerium für Verkehr.

[bibr20-0308518X251361632] Bundesministerium für Verkehr und digitale Infrastruktur (2016) Bundesverkehrswegeplan 2023. Berlin: Bundesministerium für Verkehr und digitale Infrastruktur.

[bibr21-0308518X251361632] Bundesministerium für Verkehr, Bau- und Wohnungswesen (2003) Bundesverkehrswegeplan 2003 – Grundlagen für die Zukunft der Mobilität in Deutschland. Berlin: Bundesministerium für Verkehr, Bau- und Wohnungswesen.

[bibr22-0308518X251361632] Bundesministerium für Verkehr, Bau- und Wohnungswesen (2008) Freight Transport and Logistics Masterplan. Berlin: Bundesministerium für Verkehr, Bau- und Wohnungswesen.

[bibr23-0308518X251361632] Cafruny A Ryner M (eds) (2003) A Ruined Fortress? Neoliberal Hegemony and Transformation in Europe. Lanham, MD: Rowman & Littlefield.

[bibr24-0308518X251361632] Christophers B (2011) Follow the thing: Money. Environment and Planning D: Society and Space 29(6): 1069–1084.

[bibr25-0308518X251361632] Chua C (2022) Logistics. In: Skeggs B Farris S Toscano A (eds) The Sage Handbook on Marxism. London: Sage, pp.1442–1460.

[bibr26-0308518X251361632] Chua C Danyluk M Cowen D , et al (2018) Introduction: Turbulent circulation – Building a critical engagement with logistics. Environment and Planning D: Society and Space 36(1): 617–629.

[bibr27-0308518X251361632] Clover J (2016) Riot.Strike.Riot. London: Verso Books.

[bibr28-0308518X251361632] Coe NM (2020) Logistical geographies. Geography Compass 14(10): 1–16.

[bibr29-0308518X251361632] Commission of the European Communities (1985) Completing the Internal Market- White Paper from the Commission to the European Council. Brussels: Commission of the European Communities.

[bibr30-0308518X251361632] Cook I (2004) Follow the thing: Papaya. Antipode 36(4): 642–664.

[bibr31-0308518X251361632] Cowen D (2014) Deadly Life of Logistics: Mapping Violence in Global Trade. Minneapolis, MN: University of Minnesota Press.

[bibr32-0308518X251361632] Danyluk M (2018) Capital’s logistical fix: Accumulation, globalization, and the survival of capitalism. Environment and Planning D: Society and Space 36(4): 630–547.

[bibr33-0308518X251361632] De Smedt L De Wispelaere F (2020) Road Freight Transport in the EU: In Search of a Balance Between the Economic and Social Dimension of the Internal Market. Belgium: KU Leuven, Research Institute for Work and Society.

[bibr34-0308518X251361632] Deutscher Bundestag (1973) Unterrichtung durch die Bundesregierung – Bundesverkehrswegeplan 1. Stufe. Bonn, Deutschland: Bonner Universitäts-Buchdruckerei.

[bibr35-0308518X251361632] Deutscher Bundestag (2022) ‘A100 qualifiziert beenden.’ 15 Mai. Drucksache 20/1913. Berlin: Deutscher Bundestag.

[bibr36-0308518X251361632] DGG (2024) Welcome to Deutsche GVZ-Gesellschaft mbH. Available at: https://www.gvz-org.de/en/ (accessed 15 January 2025).

[bibr37-0308518X251361632] Economic Development Agency Brandenburg (2019) Logistics in the Capital Region of Berlin-Brandenburg. Potsdam: Wirtschaftsförderung Land Brandenburg GmbH.

[bibr38-0308518X251361632] Engler H (2020) Social movement and the failure of car-friendly city projects: East and West Berlin (1970s and 1980s). The Journal of Transport History 41(3): 353–380.

[bibr39-0308518X251361632] Erne R Stan S Golden D , et al (2024) Politicising Commodification: European Governance and Labour Politics from Financial Crisis to the Covid Emergency. Cambridge: Cambridge University Press.

[bibr40-0308518X251361632] Escher F (2020) Berlin wird Metropole: Eine Geschichte der Region. Berlin: Eisengold Verlag.

[bibr41-0308518X251361632] European Commission (2009) Regulation (EC) 1072/2009 of the European Parliament and of the Council, 21 October on Common Rules of Access to the International Road Haulage Market. Brussels: European Commission.

[bibr42-0308518X251361632] European Commission (2020) Mobility Package I on Road Transport – Declaration by the Commission (2020/C 252/01). Brussels: European Commission.

[bibr43-0308518X251361632] European Parliament (2018) Directive (EU) 2018/957 of the European Parliament and Council of 28 June amending Directive 96/71/EC concerning the posting of workers in the framework of the provision of services. EUR-LEX.

[bibr44-0308518X251361632] European Transport Workers’ Federation (2023a) Third-Country Drivers in European Road Transport. Brussels: European Transport Workers Federation.

[bibr45-0308518X251361632] European Transport Workers’ Federation (2023b) Major Global Brands Implicated in Blatant Violation of Drivers’ Rights as Wildcat Strike Continues. 12 April. Available at: https://www.etf-europe.org/major-global-brands-implicated-in-blatant-violation-of-drivers-rights-as-wildcat-strike-continues/ (accessed 15 March 2025).

[bibr46-0308518X251361632] European Transport Workers’ Federation (2025) Hegelmann Group – New Evidence of Exploitation of Third-country Truck Drivers. Brussels: European Transport Workers Federation.

[bibr47-0308518X251361632] European Transport Workers’ Federation and International Transport Workers’ Federation (2023) Second Wildcat Strike in Gräfenhausen Shines New Light on Continued Human Rights Abuses in European Road Transport. 27 July. Brussels: European Transport Workers’ Federation.

[bibr48-0308518X251361632] EUROPLATFORMS (2015) EUROPLATFORMS: European Association of Transport & Logistic Centres. October. Corporate Presentation. Brussels: EUROPLATFORMS.

[bibr49-0308518X251361632] Eurostat (2008) Europe in Figures-Eurostat Yearbook 2008. Luxembourg: Eurostat.

[bibr50-0308518X251361632] Eurostat (2009) Trends in road freight transport 1999-2007- freight grew by 4% in 2007. Statistics in Focus – Transport. Luxembourg: Eurostat.

[bibr51-0308518X251361632] Federal Government (of Germany) (2017) Freight Transport and Logistics Masterplan. Berlin: Bundesministerium für Verkehr, Bau und Stadtentwicklung.

[bibr52-0308518X251361632] Global Infrastructure Hub (2023) In which infrastructure sectors are G20 governments investing? 12 April. Available at: https://www.gihub.org/infratracker-insights/in-which-infrastructure-sectors-are-g20-governments-investing/#:~:text=The%20transport%20sector%20was%20allocated,water%20and%20waste%20sectors%20combined (accessed 4 March 2025).

[bibr53-0308518X251361632] Hall P Hesse M Rodrigue J-P (2006) Re-exploring the interface between transport geography and economic geography. Environment and Planning A: Economy and Space 38(8): 1401–1408.

[bibr54-0308518X251361632] Harvey D (1990) Between space and time: Reflections on the geographical imagination. Annals, Association of American Geographers 80(3): 418–434.

[bibr55-0308518X251361632] Harvey D (1999) Limits to Capital. London: Verso Books.

[bibr56-0308518X251361632] Harvey D (2001) Spaces of Capital: Towards a Critical Geography. New York: Routledge.

[bibr57-0308518X251361632] Hesse M (2008) The City as a Terminal: The Urban Context of Logistics and Freight Transport. London: Routledge.

[bibr58-0308518X251361632] Hesse M Rodrigue JP (2004) The transport geography of logistics and freight distribution. Journal of Transport Geography 12(3): 171–184.

[bibr59-0308518X251361632] Hilal N (2008) Unintended effects of deregulation in the European Union: The case of road freight transport. Sociologie du Travail 50(1): 19–29.

[bibr60-0308518X251361632] IHK (2024a) Schule Eins Berlin Pankow 20.11.2024 – Diskussion zum Ausbau der Stadtautobahn A100. Dr.-Ing. Lutz Kaden. Berlin: Industrie und HandlesKammer zu Berlin.

[bibr61-0308518X251361632] IHK (2024b) Politische Position – 4x4 Prioritäten für eine zukunftsfähige Verkehrspolitik. Berlin: Industrie und HandlesKammer zu Berlin.

[bibr62-0308518X251361632] Interview B-5 (2024) Confidential interview with a representative from Bündnis A100 Stoppen. 23 May. Berlin, Germany.

[bibr63-0308518X251361632] Interview B-7 (2025) Confidential interview with a representative from the Gemeinde Grossbeeren GVZ. 11 February. Grossbeeren, Germany.

[bibr64-0308518X251361632] Interview B-8 (2025) Confidential interview with a representative of Ver.di (services trade union for service sector employees). 12 February. Berlin, Germany.

[bibr65-0308518X251361632] Interview B-9 (2025) Confidential interview with a representative from Transport Policy Spokesperson of the Bündnis 90/Die Grünen (Bundestag). 12 February. Berlin, Germany.

[bibr66-0308518X251361632] Interview B-10 (2025) Confidential interview with a representative of Faire Mobilität. 13 February. Berlin, Germany.

[bibr67-0308518X251361632] Interview B-12 (2025) Confidential interview with a representative of Die Bündnis/Die Grünen Berlin. 13 February. Berlin, Germany.

[bibr68-0308518X251361632] Interview B-14 (2025) Confidential interview with a representative from Die Linke in Bundestag. 14 February. Berlin, Germany.

[bibr69-0308518X251361632] Interview B-15 (2025) Confidential interview with a representative from the Autobahn GmbH. 14 February. Berlin, Germany.

[bibr70-0308518X251361632] Interview B-16 (2025) Confidential interview with a representative from the Industrie- und Handelskammer (IHK) Berlin. 15 February. Berlin, Germany.

[bibr71-0308518X251361632] Interview BRU-2 (2025) Confidential interview with a representative from a European Road Hauliers Association on 4 February. Brussels, Belgium.

[bibr72-0308518X251361632] Interview BRU-3 (2025) Confidential interview with a representative from the European Transportation Workers’ Federation. Brussels, Belgium.

[bibr73-0308518X251361632] Interview BRU-4 (2025) Confidential interview with the European Commission of Mobility and Transport. 5 February. Brussels, Belgium.

[bibr74-0308518X251361632] Interview BRU-5 (2025) Confidential interview with representatives from International Road Transport Union (IRU) on 6 February. Brussels, Belgium.

[bibr75-0308518X251361632] Investitionsbank Berlin (2024) Berlin aktuell- Berliner Aussenwirtschaft- Eine Analyse nach Länder- und Warengruppen. Berlin: Investitionsbank Berlin.

[bibr76-0308518X251361632] IRU (2023) Global Truck Driver Shortage to Double by 2028, Says New IRU Report. Brussels: IRU Permanent Delegation to the EU.

[bibr77-0308518X251361632] IRU (2024) Driving a Safe, Prosperous and Sustainable Europe – Manifesto 2024-2029 for EU Commercial Road Transport. Brussles, Belgium: IRU Permanent Delegation to the EU.

[bibr78-0308518X251361632] Kalendar U (2012) Die Geschichte der Verkehrsplannung Berlins. Köln: Forschungsgesellschaft fuer Strassen- und Verkehrswesen e.V.

[bibr79-0308518X251361632] Karapin R (2007) Protest Politics in Germany: Moements on the Left and Right since the 1960s. University Park: Penn State University Press.

[bibr80-0308518X251361632] Klauenberg J Elsner L-A Knischewski C (2020) Dynamics of the spatial distribution of hubs in groupage networks – The case of Berlin. Journal of Transport Geography 88(6): 434–502.

[bibr81-0308518X251361632] Knill C Lehmkuhl D (2002) The national impact of European Union regulatory policy: Three Europeanization mechanisms. European Journal of Political Research 41(2): 255–280.

[bibr82-0308518X251361632] McGauran K (2016) The Impact of Letterbox-Type Practices on Labour Rights and Public Renvenue. Brussels: European Trade Union Confederation (EUTC).

[bibr83-0308518X251361632] McGrath J (2021) Report on Labour Shortages and Surpluses. Luxembourg: European Labour Authority.

[bibr84-0308518X251361632] McManus P Haugton G (2021) Fighting to Undo a Deal: Identifying and Resisting the Financialization of the WestConnex Motorway, Sydney, Australia. Environment & Planning A: Economy and Space 53(1): 131–149.

[bibr85-0308518X251361632] Marx K (1990) Capital. Volume One. London: Penguin.

[bibr86-0308518X251361632] Ojala M (2020) Doing away with the sovereign: Neoliberalism and the promotion of market discipline in European economic governance. New Political Economy 26(1): 203–215.

[bibr87-0308518X251361632] Ouma S (2023) Defetishizing the asset form. Dialogues in Human Geography 14(1): 30–33.

[bibr88-0308518X251361632] Plehwe D (1998) Transformation der Logistik. Discussion Paper FS I 980 103. Berlin: Wissenschaftszentrum Berlin.

[bibr89-0308518X251361632] RBB24 (2024) Berliner StadtautobahnA100-Ausbau soll mit 1,8 Milliarden Euro nochmal deutlich teurer werden, RBB24, 18 September.

[bibr90-0308518X251361632] RBB24 (2025) Neuer Abschnitt der Berlienr A100 soll erst im September eröffnen. 28 April. Available at: https://www.rbb24.de/panorama/beitrag/2025/04/berlin-a100-verzoegerung-abschnitt-16-betrieb-ab-september-.html (accessed 5 May 2025).

[bibr91-0308518X251361632] Röber M (1991) Eine neue Verkehrspolitik für Berlin. Heft 20. Berlin: Fachhochschule für Verwaltung und Rechtspflege.

[bibr92-0308518X251361632] Rodrigue J-P (2006) Challenging the derived transport-demand thesis: Geographical issues in freight distribution. Environment and Planning A: Economy and Space 38(2): 1449–1462.

[bibr93-0308518X251361632] RTDD (2020) Pandemic of Exploitation in European Trucking: VNB-ITF-IUF Report on European Road Transport. The Netherlands: Road Transport Due Diligence (RTDD).

[bibr94-0308518X251361632] RTDD (2023) Widespread Exploitation in the EU Road Transport Industry: Th Case of Central Asian Truck Drivers. The Netherlands: Road Transport Due Diligence (RTDD).

[bibr95-0308518X251361632] Savitzky S Cidell J (2022) Whose streets? Roadway protests and weaponised automobility. Antipode 55(5): 1479–1495.

[bibr96-0308518X251361632] Senatsverwaltung für Mobilität, Verkehr, Klimaschutz und Umwelt (2021) Integriertes Wirtschaftsver-kehrskonzept Berlin. Berlin Senate: Senatsverwaltung für Mobilität, Verkehr, Klimaschutz und Umwelt.

[bibr97-0308518X251361632] Senatsverwaltung für Mobilität, Verkehr, Klimaschutz und Umwelt (2024) Verkehrsmengenkarte DTVW Kfz/Lkw 2023 – Ergebnisbericht. Berlin Senate: Senatsverwaltung für Mobilität, Verkehr, Klimaschutz und Umwelt.

[bibr98-0308518X251361632] Sheller M (2018) Mobility Justice: The Politics of Movement in an Age of Extremes. London: Verso.

[bibr99-0308518X251361632] Šimurková P Poliak M (2019) Identification of letterbox companies in the road transport sector. Transportation Research Procedia 40(2): 1184–1191.

[bibr100-0308518X251361632] Soederberg S (2021) Urban Displacements: Governing Surplus and Survival in Global Capitalism. London: Routledge.

[bibr101-0308518X251361632] Stehlin J (2023) “Freeways without futures”: Urban highway removal in the United States and Spain as socio-ecological fix? Environment & Planning E: Nature and Space 7(3): 1391–1417.

[bibr102-0308518X251361632] Sternberg HS Hofmann E Overstreet RE (2020) Perils of road freight market deregulation: Cabotage in the European Union. The International Journal of Logistics Management 31(2): 333–355.

[bibr103-0308518X251361632] The Berliner (2023) Roads to nowhere: How Berlin protest can stop the A100 extension. 25 October. Available at: https://www.the-berliner.com/politics/roads-to-nowhere-how-berlin-protest-can-stop-the-a100-extension/ (accessed 15 January 2025).

[bibr104-0308518X251361632] Tvedt J (2024) EU’s ‘three-in-seven’ road haulage cobatage rule – Impact imbalances across members states and geography. Transport Policy 159: 57–66.

[bibr105-0308518X251361632] Viscelli S (2016) The Big Rig: Trucking and the Decline of the American Dream. Oakland, CA: University of California Press.

[bibr106-0308518X251361632] Vitols K Voss E (2021) Driver Fatigue in European Road Transport. Brussels: European Transport Workers’ Federation.

[bibr107-0308518X251361632] Wahl M Weirich A (2023) Lebens- und Arbeitsbedingungen der Lkw-Fahrenden auf Parkplätzen in Deutschland: Erfahrungen aus der Beratungspraxis von Faire Mobilität. Berlin: Faire Mobilität.

[bibr108-0308518X251361632] Whyte IB Frisby D (2012) Metropolis Berlin: 1880-1940. Berkeley, CA: University of California Press.

[bibr109-0308518X251361632] Zimm A (1961) Westberlin: Die Industriestandort Westberlin under den Bedingungen der Frontstadt. Eine politisch- und ökonomisch-geographische Charakteristik. Berlin: VEB Deutscher Verlag der Wissenschaften.

[bibr110-0308518X251361632] Zimm A (1988) Berlin und sein Umland: Eine geographsiche Monographie. Gotha, Thüringen, Germany: Gotha VEB Hermann Haack.

